# Attrition of Hepatic Damage Inflicted by Angiotensin II with α-Tocopherol and β-Carotene in Experimental Apolipoprotein E Knock-out Mice

**DOI:** 10.1038/srep18300

**Published:** 2015-12-16

**Authors:** Kaliappan Gopal, Munusamy Gowtham, Singh Sachin, Mani Ravishankar Ram, Esaki M. Shankar, Tunku Kamarul

**Affiliations:** 1 Center for Cellular and Molecular Biology (CCMB), Uppal Road, 500007, Hyderabad, India; 2 Department of Bio-Medical Science, Faculty of Medicine, University of Malaya, 50603 Lembah Pantai, Kuala Lumpur, Malaysia; 3 Tropical Infectious Disease Research and Education Center (TIDREC), Department of Medical Microbiology, Faculty of Medicine, University of Malaya, 50603 Lembah Pantai, Kuala Lumpur, Malaysia; 4 Department of Orthopedic Surgery, Tissue Engineering Group (TEG), National Orthopedics Center for Excellence in Research and Learning (NOCERAL), Faculty of Medicine, University of Malaya, 50603 Lembah Pantai, Kuala Lumpur, Malaysia

## Abstract

Angiotensin II is one of the key regulatory peptides implicated in the pathogenesis of liver disease. The mechanisms underlying the salubrious role of α-tocopherol and β-carotene on liver pathology have not been comprehensively assessed. Here, we investigated the mechanisms underlying the role of Angiotensin II on hepatic damage and if α-tocopherol and β-carotene supplementation attenuates hepatic damage. Hepatic damage was induced in *Apoe*^*−/−*^mice by infusion of Angiotensin II followed by oral administration with α-tocopherol and β-carotene-enriched diet for 60 days. Investigations showed fibrosis, kupffer cell hyperplasia, hepatocyte degeneration and hepatic cell apoptosis; sinusoidal dilatation along with haemorrhages; evidence of fluid accumulation; increased ROS level and increased AST and ALT activities. In addition, tPA and *uPA* were down-regulated due to 42-fold up-regulation of *PAI-1. MMP-2, MMP-9, MMP-12,* and *M-CSF* were down-regulated in Angiotensin II-treated animals. Notably, α-tocopherol and β-carotene treatment controlled ROS, fibrosis, hepatocyte degeneration, kupffer cell hyperplasia, hepatocyte apoptosis, sinusoidal dilatation and fluid accumulation in the liver sinusoids, and liver enzyme levels. In addition, *PAI-1, tPA* and *uPA* expressions were markedly controlled by β-carotene treatment. Thus, Angiotensin II markedly influenced hepatic damage possibly by restraining fibrinolytic system. We concluded that α-tocopherol and β-carotene treatment has salubrious role in repairing hepatic pathology.

Hepatic disease is caused by various etiologies namely, viral infections, alcohol or drug ingestion, metabolic disorders, portal hypertension and autoimmunity, both in the developing and developed world^1^,^2^. Angiotensin II (Ang II) is one of the key regulatory peptides implicated in the pathogenesis of certain metabolic disorders^3^,^4^. Notably, Ang II-mediated renin–angiotensin system (RAS) has now been recognized as a key regulator of liver fibrosis^2^,^5^. Liver injury reportedly stimulates the hepatic expression of components of the RAS, playing an essential role in facilitating inflammation and deposition of extracellular matrix (ECM) proteins^2^. Hepatocellular injury is a multifunctional process involving several cell phenotypes, especially hepatic stellate cells (HSCs) and kupffer cells, cytokines, chemokines and growth factors culminating in imbalance of liver homeostasis^6^,^7^. While acute hepatocellular injury leads to transient healing responses and restoration of normal liver architecture, chronic injury results in the persistent activation of wound healing responses and accentuated accumulation of ECM proteins^2^.

Recent lines of evidence also suggest that Ang II could trigger the release of ROS^8^, leading to the onset of oxidative stress-associated diseases via activation of transcription factors, ion channels, modification of tyrosine and protein kinases/phosphatases^4^,^9^. ROS also induces apoptosis affecting the expression of certain inflammatory and ECM genes^9^. Moreover, ROS is also responsible for hepatic damage besides portal hypertension, another likely cause of hepatic degeneration^2^. It has been well established that Ang II is a precursor for induction of ROS, portal hypertension, inflammation and deposition of ECM proteins leading to organ tissue repair^3^,^4^,^8^,^9^. Therefore, therapeutic strategies targeting Ang II or controlling the precursors of tissue injury appears to be a likely therapeutic strategy in the management of Ang II/oxidative stress-mediated ailments.

Antioxidants, exemplified by α-tocopherol (vitamin E) and β-carotene appear to prevent diseases associated with oxidative stress owing to their strong free radical scavenging properties^8^. The salubrious credentials of antioxidants against cardiovascular diseases have extensively been investigated. Recently, we showed that use of antioxidants dramatically improved disease pathology following Ang II-induced abdominal aortic aneurysm (AAA) and renal damage^4^,^10^. However, to the best of our knowledge, reports on the abilities of antioxidants to protect against Ang II-induced hepatic damage have seldom been investigated. Here, we aimed to answer several research questions; we evaluated the potential of Ang II to induce hepatic damage in experimental *Apoe*^*−/−*^ mice. Subsequently, we also estimated the molecular changes, and levels of circulating inflammatory cells in Ang II-induced liver damage. The therapeutic potentials of β-carotene and α-tocopherol supplementation on hepatic degeneration in *Apoe*^*−/−*^ mice were investigated using histopathological methods. Further, we also deciphered the functional role of antioxidants on different transcriptional functional systems including the plasminogen activator and inhibitor system, peroxisome proliferators-activated receptors (*PPARα, PPAR β* and *PPARγ*) and MMPs at the pre- and post-treatments levels in degenerated liver of *Apoe*^*−/−*^ mice using standard molecular methods.

## Materials and Methods

### Animals

Our experiments were conducted according to the guidelines formulated for care and use of animals in scientific research (Indian National Science Academy, New Delhi, India) at a CPCSEA (Committee for the Purpose of Control and Supervision of Experiments on Animals) registered animal facility. The experimental protocols were approved by the Institutional Animal Ethical Committee (IAEC) at CCMB (agreement no. IAEC72/07). Apolipoprotein E (*Apoe*^*−/−*^) knockout mice were obtained from Jackson Laboratory, USA. The homogenous strains where identified by PCR using gene-specific primers. We used 4-months-old male *Apoe*^*−/−*^(n = 30) mice in our experiment and these mice were housed in accordance with the guidelines for care and use of animals. The animals were maintained in cabin type isolators under standard environmental conditions (temperature 22–25 °C and humidity 40–70%) with 12:12 dark/light (photoperiod). Mice were screened using genotype primers to differentiate homozygous from heterozygous and wild-type animals. Tail tissue samples were collected from the experimental animals and genomic DNA was isolated by phenol-chloroform method. DNA was amplified by polymerase chain reaction (PCR) using specific primer sequence recommended by The Jackson Laboratory, USA.

### Angiotensin II Treatment

Ang II was procured from Sigma-Aldrich, and ALZET osmotic pumps were from Charles River Laboratories. We infused Ang II at a dose of 1.44 mg/kg/day to experimental *Apoe*^*−/−*^ mice (n = 24) by subcutaneous administration for 45 days, and control (n = 6) animals were administered with normal saline [8]. After 45 days, Ang II-treated (n = 6) and control mice (n = 6) were sacrificed as per standard methods. The remaining Ang II-treated (n = 18) animals were administered with trans-β-carotene (n = 6) and α-tocopherol (n = 6) at a dose of 700 mg/kg of diet for 60 days, and six were left untreated, and were considered as treatment control (n = 6), which received only normal chow diet. The β-carotene and α-tocopherol supplements were mixed with rodent diet ingredients, formed to pellets (National Institute of Nutrition, Hyderabad) and fed to the animals. The diet was stored at 4^o^C and mice feed pellet were changed once in two days to minimize the oxidation and degradation of antioxidants. After 60 days, the antioxidant-treated animals and control animals were sacrificed as per standard procedures^11^.

### Biochemical Analysis of Liver Functions

Prior to euthanasia, 0.5 ml of blood was collected from each mouse by orbital sinus puncture and serum was separated by centrifugation at 1500 rpm for 15 minutes. Serum AST and ALT levels were measured by enzymatic methods using a spectrophotometer (Crest Biosystems-a Division of coral clinical systems, India) in the different treated and control groups. Activities were expressed as IUL^–1^. The measurements were done in accordance with the methods of the diagnostic kits.

### Histopathological Analysis

Complete gross and histopathological evaluations were carried out in control and experimental *Apoe*^*−/−*^ animals. After euthanasia, liver was excised and the biopsy sample was either frozen in liquid nitrogen or fixed in 10%-buffered formalin. The paraffin embedded tissue was sectioned as 5-mm sections and stained with haematoxylin and eosin stain for evaluation of pathological changes under light microscope in experimental *Apoe*^*−/−*^ mice. Masson trichrome stain was used for detecting fibrosis and architectural changes and oil-red ‘O’ for the evaluation of fatty droplets (macrovesicular or microvesicular steatosis). Cryostat sections of liver biopsy were cut (10 mm), air dried, fixed with propylene glycol and then stained with oil-red ‘O’ as per standard techniques.

### Measurement of Reactive Oxygen Species (ROS) in Liver Specimens

Harvested liver tissue samples were stored in precooled Kreb’s ringer phosphate buffer (154 mMNacl, 5.6 mM Kcl, 5.5 mM Glucose, 20.1 mM HEPESE, 25 mM NaHco3; p^H^ 7.4). Approximately, 50 mg of liver tissue was taken from each sample and lysates were prepared in precooled 0.25M sucrose solution using standard procedure. The protein concentrations were estimated using the Bradford assay and final protein concentrations were made to 2 mg/ml in each sample. The release of hydrogen peroxide (H_2_O_2_) from liver tissue lysate was determined using Amplex Red reagent, using the reagents and protocols provided in the Amplex Red Hydrogen Peroxide Assay Kit (Cat. No A12222, Invitrogen). Two hundred micrograms of experimental and control sample lysates were re-suspended in 100 μl of Kreb’s Ringer phosphate buffer (p^H^ 7.4), and the assay was initiated by adding 100 μl of Kreb’s Ringer buffer solution containing 50 μM Amplex Red reagent (Cat. No A12222, Invitrogen) together with 0.1 U/mL horseradish peroxidase (Mouse Peroxidase Conjugate 595-414, LotMC1344, Xpress Bio). Instant formation of resorufin fluorescence was measured using a EnSpire multimode plate reader (Perkin Elmer, USA, EnSpire 2300, multimode Reader softwere version 4.1) at 540 nm excitation and 595 nm emissions wave length.

### Confocal Microscopy

The frozen sections (15 μm) of treated and control aorta were fixed with acetone for 20 minutes followed by permeabilization with 0.5% (v/v) Triton X-100 for 10 minutes at room temperature. Following blockade with 5% fetal calf serum (FCS) for 1 hour, the sections were incubated with mouse monoclonal antibody Mac3 (CD107b) (Cat No. 553323, BD pharmingen), CD4 (Cat No. 553729, BD Pharmingen), K19 (BD Pharmingen 550951), caspase 3 (Cat No. 271759, Santa Cruz), p53 (Cat No. Sc-56182, Santa Cruz) for 1 hr at room temperature. The sections were subsequently washed three times with 1% PBS and incubated with anti-mouse secondary antibody conjugated with FITC for 1 hour. To reduce auto-fluorescence, the sections were treated with CuSO_4_ (10 mM) in ammonium acetate buffer (50 mM, pH 5.5) for 30 minutes. The sections were counterstained with propidium iodide (PI) for 5 minutes and mounted in vector shield (Vector Laboratories, CA, USA). Confocal laser scanning immunofluorescence microscopy (CLSM) was carried out using a Zeiss LSM 510 META confocal microscope. Image analysis was done using LSM510 META software (Carl Zeiss, USA).

### cDNA Synthesis and Quantitative Real-Time PCR

To quantify the relative levels of candidate gene expressions, liver samples of Ang II, β-carotene and control mice were homogenized and total RNA was extracted using TRizol reagent (Invitrogen). The total RNA was measured using spectrophotometer (NanoDrop-1000) at 260 nm. Each sample was normalized to 2 μg of total RNA, and cDNA synthesis was performed (SuperScript^®^ III Reverse Transcriptase) using the first-strand cDNA synthesis kit (Invitrogen Corp USA.) primed with oligo(dT)20 in a 20 μL reverse transcription reaction on a Effendorf-master thermocycler. The cDNA prepared served as the template for the qRT-PCR investigations using SYBR Green PCR kit and a 7800 Sequence Detection System (Applied Biosystems). All gene specific primers were selected using gene tool software to amplify a 150–200 bp product with a Tm of 55–60 °C. The primers used were tabulated and showed in Table 1.

### Statistical Analysis

The data was determined as mean ± SD where appropriate. Comparisons and correlations of treated and control samples were made with ANOVA and paired t test. Statistical analysis was performed with GraphPad Prism 5 software (GraphPad, Inc., La Jolla, CA, USA). P values of <0.05 were considered significant.

## Results

### Subcutaneous Angiotensin-II Injection Resulted in the Induction of Hepatic Damage in *Apoe* ^*−/−*^ Mice

To investigate if Ang II infusion results in hepatic damage, we subcutaneously administered *Apoe*^*−/−*^ mice with Ang II and found that liver damage was induced by Ang II. We also determined the salubrious potentials of β-carotene and α-tocopherol against hepatic damage induced by Ang II. Ang II (50 μg/30 gm body weight of mouse) was infused subcutaneously to experimental groups for 45 days. Control animals were fed with standard diet. After 45 days of Ang II or saline infusion, the animals were euthanized (as per study design), and liver tissues and blood samples were collected to examine liver damage, and were subjected to molecular and histopathological investigations. We observed notable liver damage in the Ang II-treated group. The Ang II-treated animals in the second and third groups were fed with standard diet enriched with α-tocopherol and β-carotene in order to examine the therapeutic effects of antioxidants on liver damage, and the fourth group with standard diet for two months (60 days). The daily intake of antioxidant was ~0.008 mg/day. After two months of α-tocopherol and β-carotene treatment, the animals were euthanized, and blood and liver tissues were collected for laboratory investigations. Histopathological, biochemical, flow cytometric, immunofluorescence and qRT-PCR mRNA investigations were suggestive that antioxidant treatment could have a beneficial role in recovering the liver damage caused by Ang II treatment.

### Histopathological and Masson Trichrome Staining Analysis Showed Evidence of Angiotensin II-Induced Severe Liver Damage in Experimental *Apoe* ^*−/−*^ Mice

**Figure 1:**
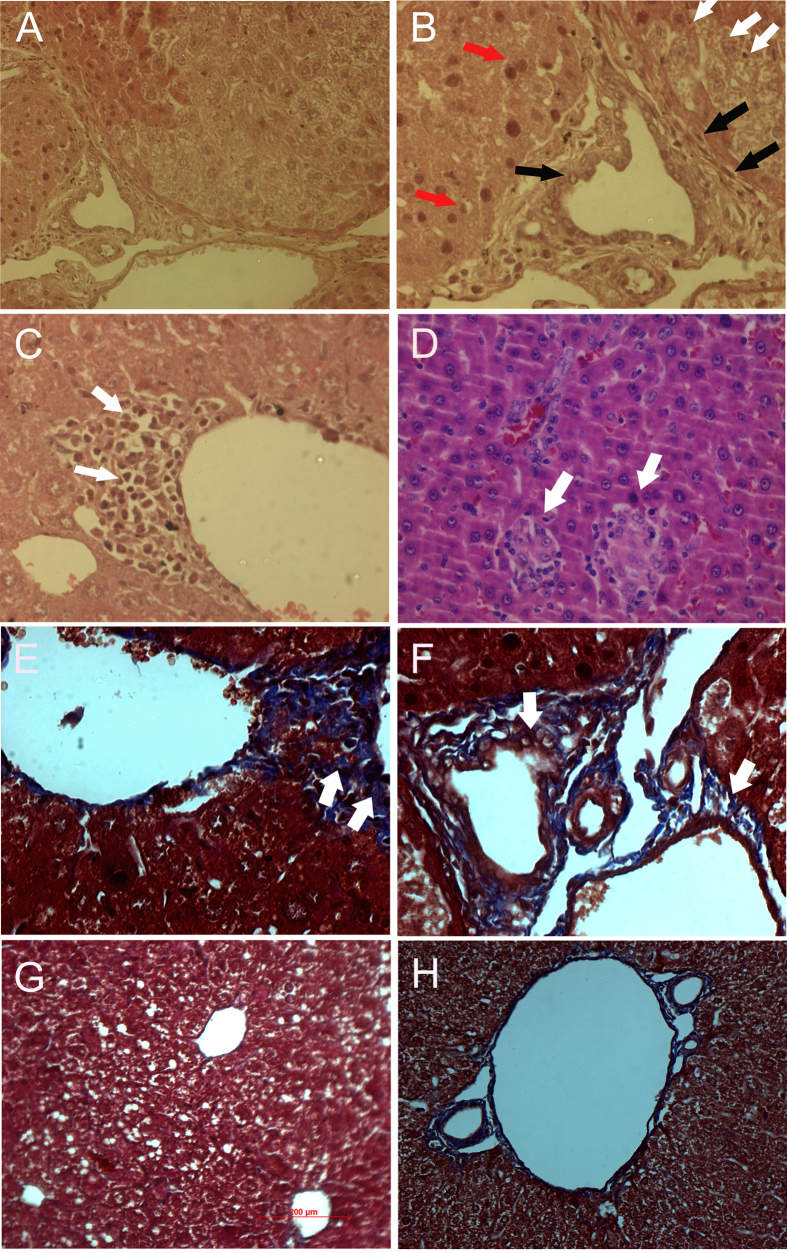
Histopathological analysis of pathological changes in the liver of Angiotensin-II- treated *Apoe*^*−/−*^ mice using Hematoxylin & Eosin and Masson trichrome staining methods. (**A,B**) Individual hepatocyte degeneration (white arrow), mild bile duct hyperplasia, periductular/peri billary fibrosis (black arrow) and few foci of individual hepatocyte apoptosis (red arrow) seen in the liver of Angiotensin-II-treated *Apoe*^*−/−*^ mice. (**C,D**) Hepatic necrosis with infiltration of inflammatory cells (white arrow); (**E,F**) Peribiliary and/or periportal fibrosis shown by Masson trichrome staining in Angiotensin-II- treated *Apoe*^*−/−*^ mice (arrow); (**G**) Liver tissue stained with Masson trichrome of β-carotene-treated *Apoe*^*−/−*^ mice lacking evidence of peribiliary or periportal fibrosis; (**H**) Control liver not showing any fibrosis.

Ang II liver showed evidence of individual hepatocyte degeneration (white arrow), mild bile duct hyperplasia, periductular and/or peribiliary fibrosis (black arrow), and few foci of individual hepatocyte apoptosis (red arrow) in the liver sections of Ang II-infused experimental mice (Fig. 1A,B). Hepatic necrosis with infiltration of inflammatory cells was also observed in the Ang II-infused experimental animals (white arrow) (Fig. 1C,D). In addition, periductular fibrosis was confirmed by Masson trichrome staining. Peribiliary and/or perifibrosis was clearly observed where collagen tissues surrounding the bile ducts assumed blue (arrow) and nucleus of bile duct stained dark red by Masson trichrome staining in the liver tissue of Ang II-treated *Apoe*^*−/−*^ mice (Fig. 1E,F). The liver tissue also showed evidence of periportal inflammation and fibrosis. Blue collagen spreading over the periportal region showed indications of fibrosis (arrow) in this region. However, liver tissue of β-carotene-treated *Apoe*^*−/−*^ mice did not show peribiliary or periportal fibrosis (Fig. 1G) as compared to the controls (Fig. 1H).

### Histological Analysis of Vascular Changes in the Liver of Angiotensin II-Treated, α-Tocopherol and β-Carotene-Treated *Apoe* ^*−/−*^ Mice

**Figure 2:**
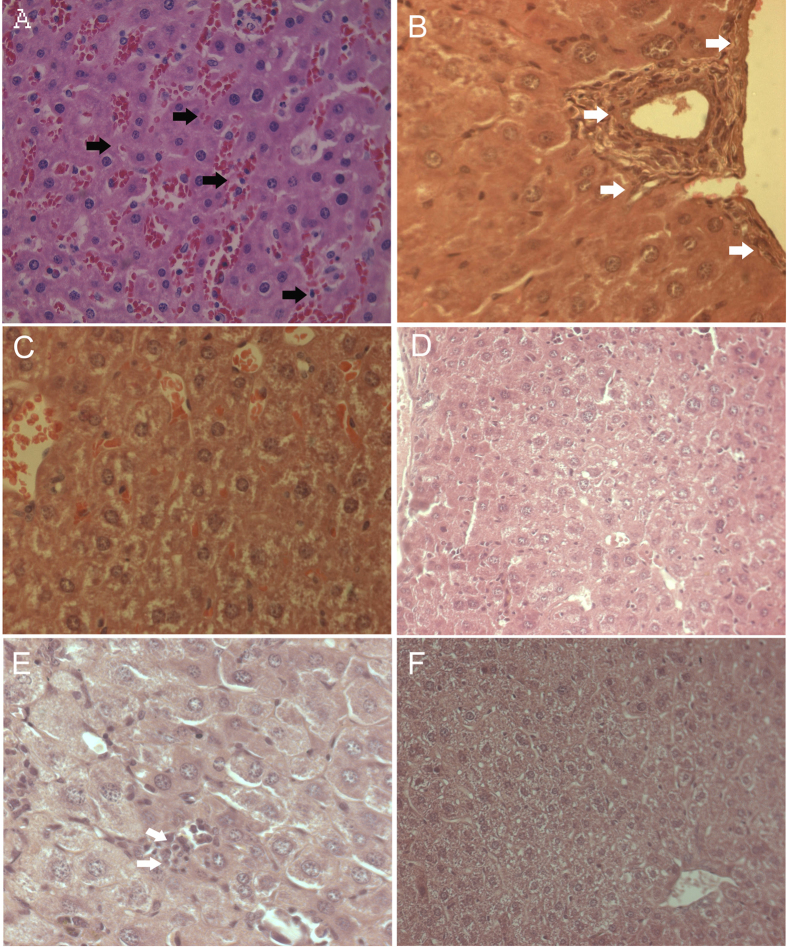
Histological analysis of vascular changes in the liver of Angiotensin-II-treated, α-tocopherol and β-carotene-treated *Apoe*^*−/−*^ mice. (**A,B**) Moderate to severe sinusoidal dilatation along with haemorrhages (black arrows) and mild to moderate inflammatory reactions where periportal/ peribillary fibrosis (white arrows) seen in Angiotensin-II-treated *Apoe*^*−/−*^ mice. (**C,D**) Vascular and inflammatory changes were not observed in β-carotene-treated *Apoe*^*−/−*^ mice. (**E,F**) Lack of fibrotic and vascular changes, with mild Kupffer cell proliferation in α-tocopherol-treated *Apoe*^*−/−*^ mice.

**Figure 3:**
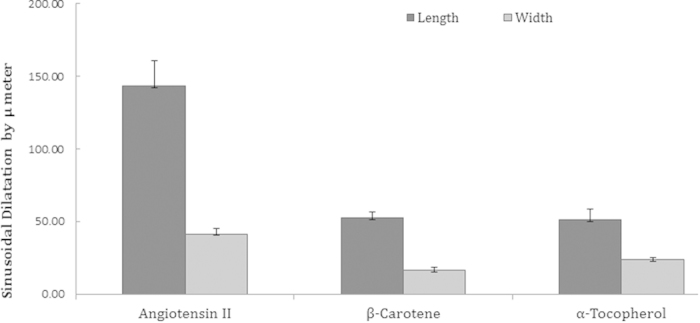
Sinusoidal dilatation in the angiotensin II-treated experimental animals and compared to β-carotene and α-tocopherol treated *Apoe*^*−/−*^ mice.

Next, we investigated for vascular changes in the liver tissues of Ang II-treated *Apoe*^*−/−*^ mice by histopathological examinations. We found notable vascular changes in the liver of the *Apoe*^*−/−*^ mice. Moderate to severe sinusoidal dilatation along with haemorrhages (black arrows) and mild to moderate inflammatory reactions, where periportal/peribiliary fibrosis (white arrows) were noticed in the Ang II-treated experimental *Apoe*^*−/−*^ mice (Fig. 2A,B). Interestingly, such vascular and inflammatory lesions were not found in the β-carotene-treated *Apoe*^*−/−*^ mice (Fig. 2C,D). Similarly, in the α-tocopherol-treated *Apoe*^*−/−*^ we did not see any fibrotic and vascular changes, although mild kupffer cell proliferation was noticed (Fig. 2E,F). In addition, we quantified the sinusoidal dilatation and found significantly increased length and width of sinusoidal spaces in the Ang II-treated experimental animals as compared to β-carotene and α-tocopherol treatment animals (Fig. 3).

### α-Tocopherol and β-Carotene Dietary Supplementation Alleviated Liver Pathology in Experimental *Apoe* ^*−/−*^ Mice

**Figure 4:**
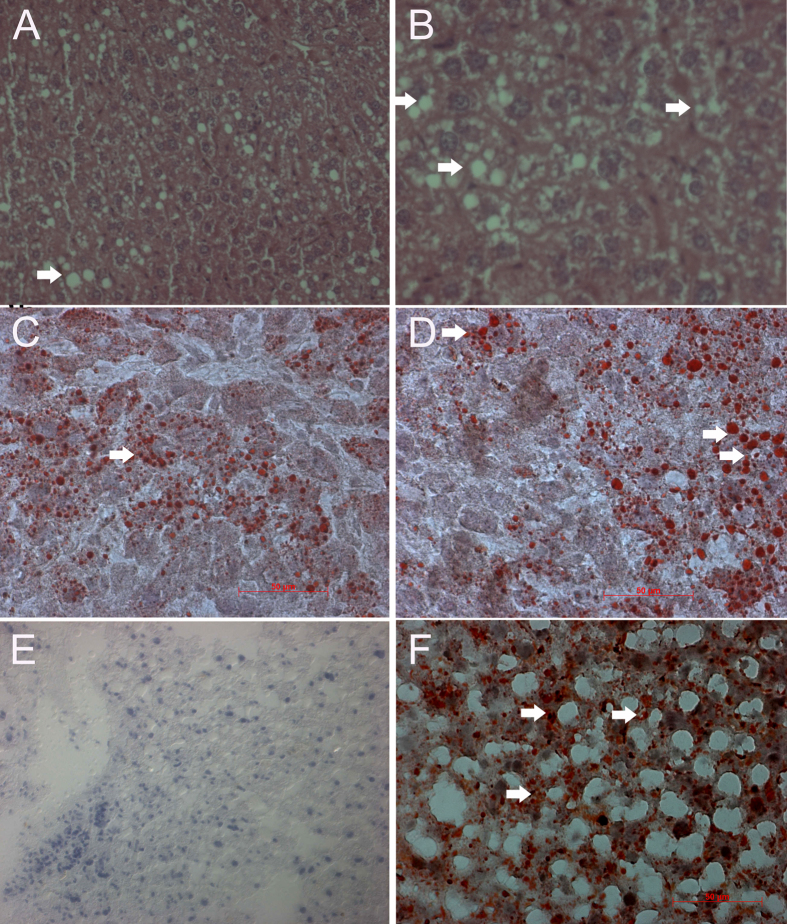
Oil-red ‘O’ staining for pathological examination of liver of α-tocopherol- and β-carotene-treated *Apoe*^*−/−*^ mice. (**A,B**) Moderate vacoular degeneration (**arrow**) in the hepatocytes of β-carotene-treated *Apoe*^*−/−*^ mice. (**C,D**) β-carotene-treated liver show moderate accumulation of red colour lipid droplets in the cytoplasm of hepatocytes (arrow). (**E**) α-tocopherol-treated liver lacking accumulation of lipid droplets as compared to positive control high fat diet-treated liver of *Apoe*^*−/−*^ mice. (**F**) Accumulation of red colour lipid droplets in the cytoplasm of hepatocytes in high fat diet-treated liver of *Apoe*^*−/−*^ mice (**arrow**) (positive control).

Next, we investigated the effect of α-tocopherol and β-carotene dietary supplementation on liver pathology in experimental *Apoe*^*−/−*^ mice by histopathological and oil-red O examinations. Interestingly, our investigation showed that α-tocopherol and β-carotene dietary supplementation markedly alleviated the liver pathology in the *Apoe*^*−/−*^ mice. The bile duct appeared normal and no hepatic necrosis or individual hepatic necrosis was observed in β-carotene experimental *Apoe*^*−/−*^ mice. However, moderate vacoular degeneration was observed in the hepatocytes of β-carotene-treated experimental *Apoe*^*−/−*^ mice (Fig. 4A,B). This was evaluated by oil-red O staining, and showed accumulation of lipid droplets in the cytoplasm of hepatocytes in liver tissue of β-carotene-treated *Apoe*^*−/−*^ mice (Fig. 4C,D). This observation is suggestive that the moderate degeneration observed could be due to fatty degeneration in the liver. Noticeably, α-tocopherol-treated liver (Fig. 4E) lacking accumulation of lipid droplets as compared to positive control high fat diet-treated livers of *Apoe*^*−/−*^ mice (Fig. 4F). Of note, the salubrious effects were clearly evident in the α-tocopherol-treated mice. Here, hepatocytes appeared normal from the degenerated stage. Mild proliferation of kupffer cells was noticed in the α-tocopherol-treated livers. However, the abnormalities observed in the Ang II-treated mice showed individual cell apoptosis, necrosis, bile duct and hyperplasia/proliferation of bile duct, which however appeared normal in the α-tocopherol-treated *Apoe*^*−/−*^ mice.

### Confocal Microscopy Analysis for Expression of Mac-3 in Angiotensin II Administration in *Apoe* ^*−/−*^ Mice

**Figure 5:**
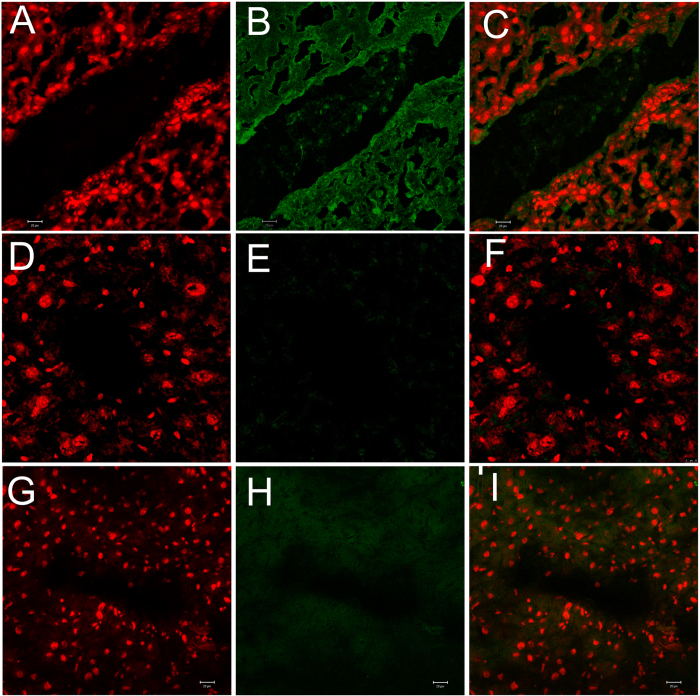
Confocal microscopy analysis of Mac3 expression in Angiotensin-II and β-carotene-treated *Apoe*^*−/−*^ mice. (**A–C**) Mac3 over expressed marginally in the liver tissues of Ang II- treated *Apoe*^*−/−*^ mice; (**D–F**) Mac3 show no expression in β-carotene-treated *Apoe*^*−/−*^ mice; (**G–I**) No expression of Mac3 is noticed in control liver.

We recently showed that macrophage and lymphocyte infiltration could play a crucial role in facilitating the inflammation in Ang II-mediated pathology involving the aorta, brain and kidney^4^,^10^,^11^,^12^. Therefore, here we investigated the potential role of macrophages and CD4 + cells in Ang II-induced hepatic damage in *Apoe*^*−/−*^ mice. Our investigations showed that Mac3 expression was increased marginally in the liver tissues of mice administered with Ang II relative to that of control mice (Fig. 5A–C). Mac3 + macrophages seen in the livers of Ang II-treated animals were largely controlled by β-carotene treatment (Fig. 5D–F) relative to control animals that showed no Mac3 expression (Fig. 5G–I). We observed only mild expression of CD4 molecules in the liver tissues of inflamed liver and no expression in the antioxidant-treated animals (data not shown).

### Confocal Microscopy Analysis of K19, p53 and Caspase 3 Expressions in Angiotensin II- and β-Carotene-Treated *Apoe* ^*−/−*^ Mice

**Figure 6:**
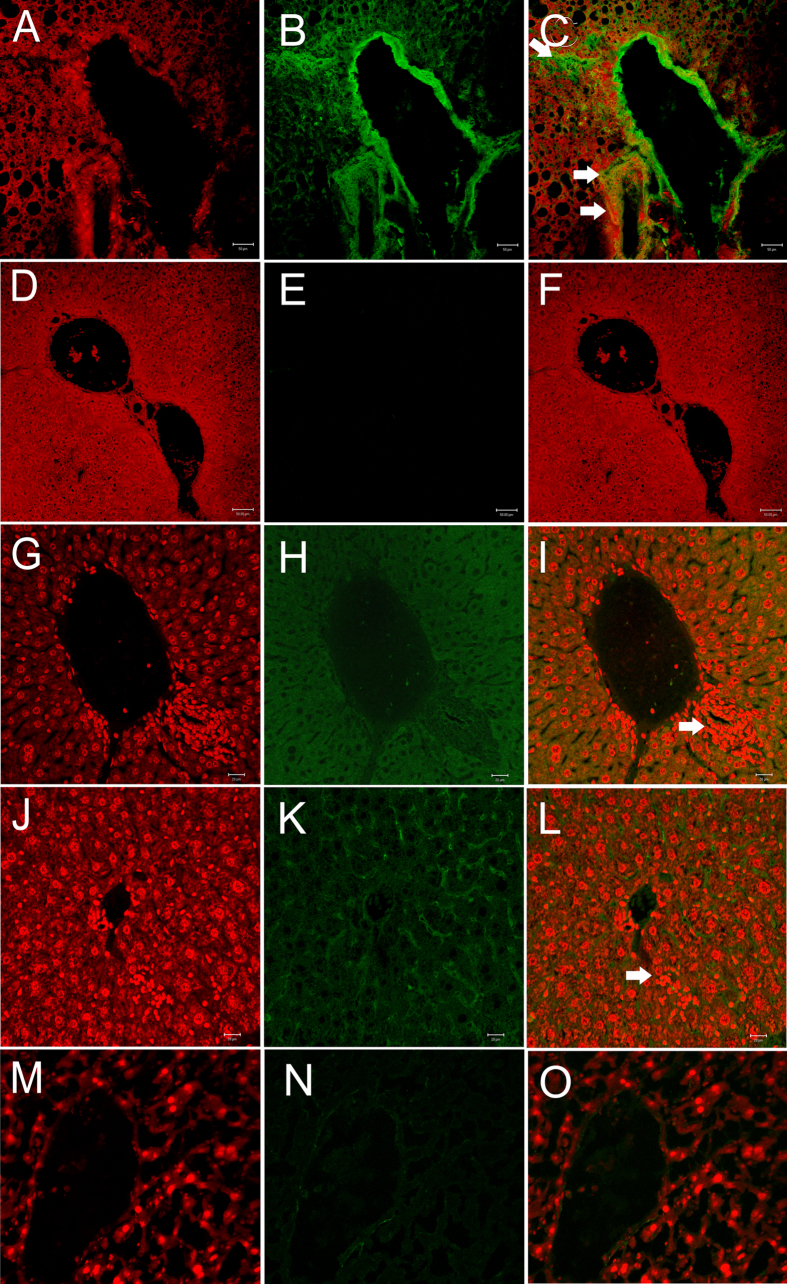
Confocal microscopy analysis of K18, p53 and caspase 3 expressions in Angiotensin-II and β-carotene-treated *Apoe*^*−/−*^ mice. (**A–C**) Cytokeratin 18 over-expression (arrow) in Angiotensin-II-treated as compared to (**D–F**) β-carotene-treated *Apoe*^*−/−*^ mice; (**G–I**) Caspase 3 over-expressed moderately in the inflammatory regions of Angiotensin-II-treated *Apoe*^*−/−*^ mice (arrow); (**J–L**) p53 show mild over-expression in the inflammatory regions (white arrow) of Angiotensin-II-treated *Apoe*^*−/−*^ mice as compared to β-carotene-treated *Apoe*^*−/−*^ mice (**M-O**).

Next, we sought to perform immunohistochemical staining with CK-19 to identify ductal proliferation, and caspase 3 and p53 to be able to differentiate necrotic versus apoptotic hepatocytes in experimental *Apoe*^*−/−*^ mice. Investigations showed the over-expression of CK-19 in Ang II-treated as compared to β-carotene-treated *Apoe*^*−/−*^ mice (Fig. 6A–F). Both Caspase 3 and p53 were moderately over-expressed in the inflammatory regions of Ang II-treated as compared to β-carotene-treated *Apoe*^*−/−*^ mice (Fig. 6G–O)

### Angiotensin II Treatment Increased the Levels of Liver Transaminases, and Antioxidant Treatment Led to Marked Recovery of Liver Damage

**Figure 7:**
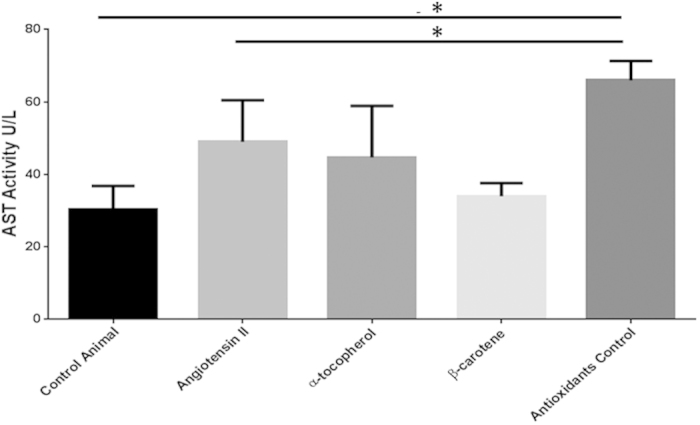
AST activity in different treatment groups of *Apoe*^*−/−*^ mice. Determined AST activity in the different treatment groups were compared with control *Apoe*^*−/−*^ mice. AST activity levels of Angiotensin-II-treated group were remarkably elevated as compared to controls. AST activity levels in the antioxidant control group (Angiotensin-II infused and no antioxidant supplements) were significantly elevated as compared to the Angiotensin-II and control groups. Footnotes: *Indicates significant difference between the individual treatment groups marked by arrow line.

**Figure 8:**
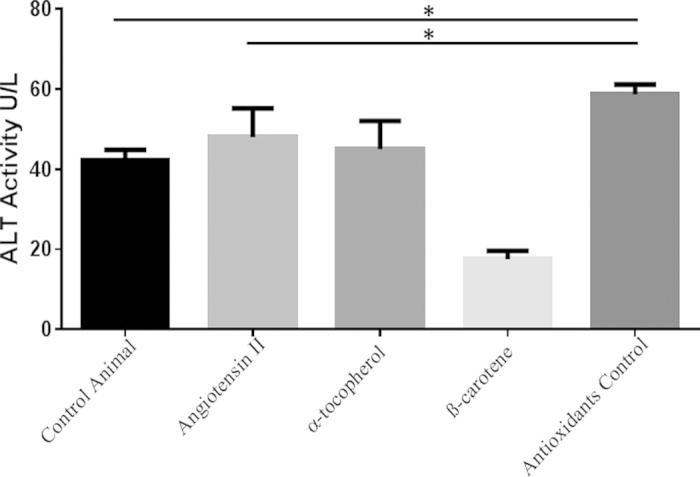
ALT activity in different treatment groups of *Apoe*^*−/−*^ mice. Examined ALT activity in the different treatment groups were compared with control *Apoe*^*−/−*^ mice. ALT activity levels of Angiotensin-II-treated group were remarkably elevated as compared to control (p~0.007). AST levels of antioxidants control group (Angiotensin-II infused and no antioxidant supplements) were significantly further elevated as compared to the Angiotensin-II and control groups. Footnotes: *Indicates significant difference between the individual treatment groups marked by arrow line.

Ongoing inflammation is marked by increase of liver enzyme levels. Our investigations showed markedly increased liver enzyme levels, AST and ALT in the serum of Ang II-infused animals relative to control animals (Table 2). Particularly, AST levels were significantly increased in the Ang II-treated group (p = 0.01) (Table 2). In addition, we also found an increase in the AST/ALT ratio among Ang II-treated animals as compared to the control mice (Table 3). We also established that β-carotene treatment largely controlled the levels of both AST and ALT in the treatment groups and no significance variation was evident between the Ang II-treated and control mice. Importantly, the treatment control animals, i.e. Ang II-treated animals fed with only standard diet without α-tocopherol and β-carotene treatment, showed AST and ALT worsened levels and significantly increased levels as compared to the Ang II-treated animals and control animals (Figs 7 and 8). This observation strongly suggests that both α-tocopherol and β-carotene treatment has significant role in the recovery of liver injury.

### Angiotensin-II Inflicted ROS levels were Alleviated by β-Carotene Supplementation

**Figure 9:**
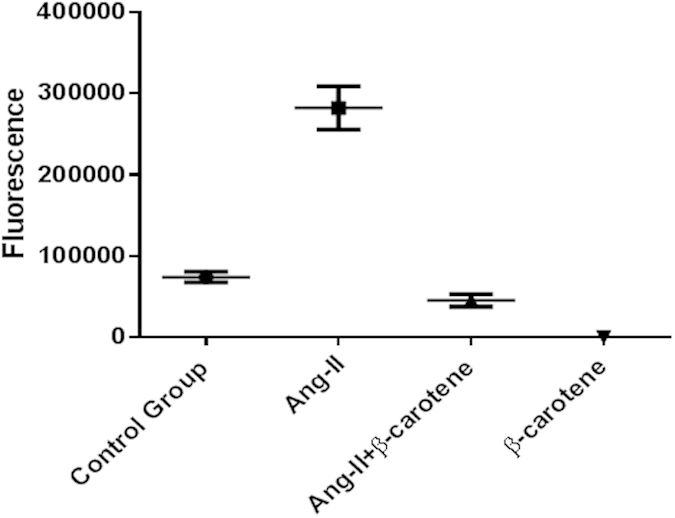
Angiotensin-II inflicted ROS levels controlled by β-carotene in *Apoe*^*−/−*^ mice. Determined ROS release in the liver tissues of different treatment groups were compared with control *Apoe*^*−/−*^ mice, and found significant difference between the treatment groups (p~0.0001). ROS releases were significantly increased in the livers of Ang-II infused *Apoe*^*−/−*^ mice as compared to control *Apoe*^*−/−*^ mice (p~0.002). ROS releases were significantly controlled in the β-carotene supplement treated *Apoe*^*−/−*^ mice as compared to Ang-II infused (p~0.002) and control *Apoe*^*−/−*^ mice (p~0.001). Footnotes: *Indicates significant difference between the individual treatment groups marked by arrow line.

Next, we determined the release of ROS in the different experimental animal liver tissues. Our investigations showed that ROS release was significantly increased in the liver of Ang-II infused *Apoe*^*−/−*^ mice as compared to the control animals. We also determined the salubrious role of β-carotene on ROS release, and we found that ROS release was significantly controlled in β-carotene treated *Apoe*^*−/−*^ mice as compared to Ang-II infused and control mice. In addition, ROS release was significantly lessened in Ang-II infused and concurrently β-carotene supplemented experimental *Apoe*^*−/−*^ mice (Fig. 9)

### Liver Damage Following Angiotensin II Administration Resulted by Influencing the Fibrinolytic Pathway

Also, we examined the mRNA levels of members of the fibrinolytic system in the liver. Increased *PAI-1* activity is reportedly associated with an increased risk of ischemic cardiovascular events and tissue fibrosis. Here, we analyzed *PAI-1*, a major physiological inhibitor of tissue type (*tPA*) and urokinase type plasminogen activators (*uPA*). The plasminogen activators of *tPA* and *uPA* were down-regulated in liver, and its inhibitor *PAI-1* were highly (42-folds) up-regulated relative to control group (Table 3). Remarkably, matrix metalloproteinase enzymes, namely *MMP-2, MMP-3, MMP-9* and *MMP-12* were several folds down-regulated in the liver of *Apoe*^*−/−*^ mice. *PAI-1*, *tPA*, *uPA* and MMPs are reportedly the key mediators in fibrinolytic pathway implicated in ECM remodelling and degradation. Our observations of *PAI-1* up-regulation, *tPA* and *uPA* controlled expression followed by MMPs down-regulation suggest the potential association of fibrinolytic pathway in Ang II-induced liver pathology.

### Expression of Adhesion Molecules and Cytokines in Damaged Liver

Several lines of investigations have shown that adhesion molecules, *ICAM-1* and *VCAM-1* could function as key pathologic mediators of vascular diseases. Our current investigations showed that *ICAM-1* and *VCAM-1* were slightly down-regulated in the Ang II-treated livers. The mRNA level of *M-CSP-1* was 52-folds down-regulated (very highly) in the livers of *Apoe*^*−/−*^ mice as compared to control animals (Table 4).

### α-Tocopherol Treatment Systematically Controlled Fibrinolytic Pathway in the Livers of *Apoe* ^*−/−*^ Mice

**Figure 10:**
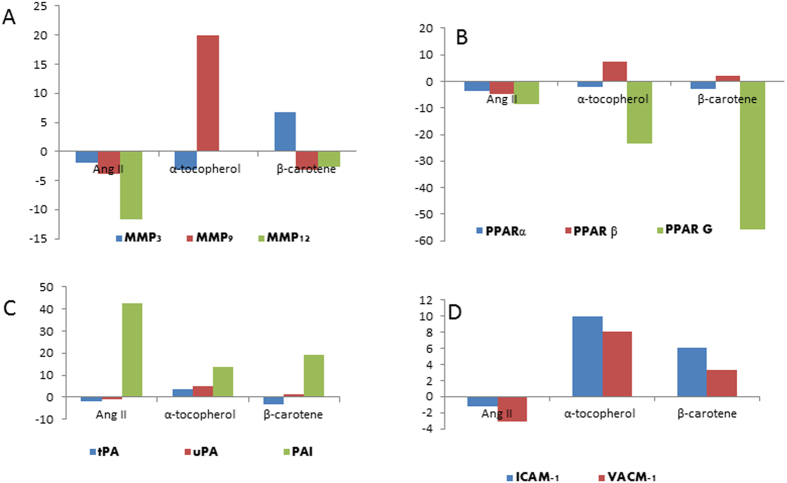
Fold changes of selective candidate genes mRNA level in Angiotensin-II-infused, and α-tocopherol and β-carotene-treated *Apoe*^*−/−*^ mice. (**A**) MMP-3, MMP-9 and MMP-12 fold gene expression. MMP-12 down-regulated in the liver of Angiotensin-II treated group; (**B**) PPAR-α, PPAR-β and PPAR-γ down-regulated in Angiotensin-II- and both α-tocopherol- and β-carotene-treated animals; (**C**) PAI-1 up-regulated, and both tPA and uPA down-regulated in the Angiotensin-II-treated mice. α-tocopherol- and β-carotene-treated animals controlled PAI-1 expression and improved the levels of both tPA and uPA mRNA. (**D**) ICAM-1 and VCAM-1 down-regulated in the Angiotensin-II-treated group and up-regulated in both α-tocopherol- and β-carotene-treated *Apoe*^*−/−*^ mice.

Next, we set out to investigate if α-tocopherol treatment systematically controlled the fibrinolytic pathway in the liver of *Apoe*^*−/−*^ mice. Our studies showed that *PAI-1* was greatly controlled by α-tocopherol (13-folds) in the liver of Ang II-treated mice relative to the controls. Further, the plasminogen activators of *tPA* and *uPA* were up-regulated to 4.9 and 3.6-folds respectively in the α-tocopherol treatment group. We also showed that *MMP2, MMP9* and *MMP12* were up-regulated by α-tocopherol treatment in *Apoe*^*−/−*^ mice. In addition, adhesion molecules *ICAM-1* and *VCAM-1* were up-regulated by both the antioxidants. Notably, *MCP-1* was upregulated to 2-fold in both α-tocopherol and β-carotene-treated animals (Fig. 10). Further, we also deciphered the functional role of antioxidants on peroxisome proliferators-activated receptors (*PPARα, PPARβ* and *PPARγ*) at both pre- and post-treatments levels in degenerated liver of *Apoe*^*−/−*^ mice. However, investigation showed no significant difference in the treated animals as compared to control *Apoe*^*−/−*^ mice, and these genes were markedly down-regulated in the Ang II-treated animals and antioxidant-treated animals relative to the control animals.

## Discussion

Several diseases have underpinned the association of the RAS pathway with Ang II-mediated tissue injury^4^,^10^,^12^,^13^. However, high resolution studies are seldom available on Ang II-induced liver damage heretofore. The results of the present study indicate that increased levels of Ang II cause hepatic damage by triggering the hyperplasia of kupffer cells, hepatocyte degeneration and hepatocyte apoptosis, sinusoidal dilatation along with haemorrhages and sinusoids showing fluid accumulation in the liver. Further, numerous inflammatory Mac3 + macrophages were observed in the liver of Ang II-treated animals. In addition, biochemical indicators of active liver inflammation namely AST and ALT were increased, which strongly supports this conclusion^14^. Notably, treatment control animals fed with only standard diet without antioxidants (α-tocopherol and β-carotene), showed significantly increased AST and ALT levels compared to Ang II-treated animals and control group animals. This observation strongly suggests that increased Ang II levels could largely impact hepatic pathogenesis in the experimental *Apoe*^*−/−*^ mice.

Several studies suggest that antioxidants could be used as potential therapeutic agents against diseases associated with oxidative stress mediated by Ang II, although evidence supporting these suggestions is equivocal^4^,^10^,^12^. α-tocopherol is a major fat-soluble antioxidant found in lipid-phase membranes. Several studies have clearly shown that serum levels of α-tocopherol are significantly decreased in patients with alcoholic liver disease. Vitamin E can facilitate scavenging of free radicals generated in liver tissues^15^. Pre-treatment with vitamin E reportedly reduced the degree of oxidative stress^16^. In the mouse model, vitamin E supplementation restored alcohol-induced redox status, reduced apoptosis, and prevented oxidative stress. In addition, vitamin E in doses of 600 mg daily was found to be effective in suppressing HBV replication and normalizing ALT in a significant proportion of chronically infected patients with chronic liver disease^15^,^17^. Our current investigation finds significant effect of antioxidants on Ang II-induced liver injury. Notably, we observed relatively lesser hepatocyte degeneration and apoptosis, kupffer cell hyperplasia, sinusoidal dilatation along with haemorrhages and sinusoids in concert with accumulation of fluid in the liver in both the antioxidant-treated viz., α-tocopherol and β-carotene treatment groups. Further, recruitment of inflammatory Mac3 + macrophages was largely controlled in the β-carotene-treated group. In addition, well evidence that Ang-II could trigger intracellular accumulation of ROS, which is involved in myriad of downstream signaling pathways viz., transcription factors, tyrosine kinases, protein kinases, ion channels and certain other potential molecular targets. Moreover, ROS is also responsible for hepatic damage besides portal hypertension and hepatic degeneration. Our current investigations showed increased release of ROS in the liver of Ang-II infused *Apoe*^*−/−*^ mice, further supporting that Ang-II could cause hepatic damage by triggering intracellular accumulation of ROS. Interestingly, our investigation also found that ROS release was significantly controlled by β-carotene dietary supplementation. This salubrious role of β-carotene treatment in the *Apoe*^*−/−*^ mice reflects the beneficial role of antioxidants^11^,^18^ against hepatic damage.

Activated kupffer cells, infiltrating monocytes, activated and aggregated platelets, and damaged hepatocytes are believed to be the sources of platelet-derived growth factor (PGF) and TGF-β, which triggers the initiation of intracellular signaling cascades leading to activation of HSCs. Further, HSC activation is common to all forms of liver injury and fibrosis^6^. Moreover, previous studies also suggest that cytokines may be implicated in the recruitment of circulating macrophages into the liver and activation of kupffer cells and HSCs, both contributing to progression from simple steatosis to steatohepatitis^6^,^7^. Therefore, considering present treatment effects on hepatic liver, α-tocopherol and β-carotene attenuates or prevents the progression of severe hepatic damage by controlling the infiltration of inflammatory cells.

Importantly, serum AST and ALT levels were significantly increased in Ang II-infused liver. Further, β-carotene treatment greatly controlled the both liver enzyme levels in the treated groups. Importantly, treatment control group i.e. Ang II-treated animals fed only with standard diet without α-tocopherol and β-carotene, showed worsened levels of ALT and AST, and were significantly increased compared to Ang II-treated animals and control group animals (p = 0.02). This observation strongly suggests two points (i) increased Ang II levels highly influences chronic hepatic pathogenesis; (ii) Both α-tocopherol and β-carotene treatment has significant beneficial role in the recovery process of liver injury. These observations strongly indicate that β-carotene and α-tocopherol has significant treatment effect on Ang II-induced liver pathology. AST/ALT ratio is one of the common parameters used in the diagnosis of liver fibrosis^14^. Our current study showed that Ang II-treated group and the treatment control group animals showed an increased AST/ALT ratio of >1 suggesting the possibility to progression of liver fibrosis. We also observed that the control group and α-tocopherol-treated group showed an AST/ALT ratio of <1. Earlier studies on chronic liver disease of various etiologies—non-alcoholic liver, chronic viral hepatitis, primary sclerotizing cholangoitis, and primary biliary cirrhosis and a few other large scale studies suggest that AST/ALT ratio >1 often means that excessive amount of fibrous tissue has accumulated in the liver^14^,^19^,^20^.

Current investigation examined the mRNA levels of fibrinolytic system regulatory genes in the liver, which showed that the plasminogen activators of *tPA* and *uPA* were markedly down-regulated in the liver, and its inhibitor *PAI-1* was enormously up-regulated (42-fold) as compared with the control group. Remarkably, MMP enzymes that include *MMP2, MMP3, MMP9* and *MMP12* were several folds down-regulated in the livers of *Apoe*^*−/−*^ mice. *PAI-1, tPA, uPA* and *MMPs* are reportedly important mediators in the fibrinolytic pathway implicated also in ECM remodelling and degradation. *PAI-1* up-regulation, *tPA* and *uPA* controlled expression followed by MMPs down-regulation suggest the potential involvement of the fibrinolytic pathway in the disease pathogenesis of Ang II-associated liver injury. Recent evidence suggests that increased *PAI-1* activity could be associated with an increased risk of hepatic fibrosis^21^. The fibrinolytic system comprises an inactive pro-enzyme, plasminogen that can be converted to active plasmin, which degrades fibrin and activates MMPs, which in turn degrades extracellular matrix^12^. An increased accumulation of extracellular matrices could lead to liver fibrosis^22^. *PAI-1* is a major inhibitor of both *tPA* and *uPA* and therefore, is a key regulator of fibrinolysis by plasmin^21^. In addition to regulating the accumulation of fibrinogen/fibrin in the extracellular space, plasmin could also directly degrade other ECM components such as laminin, proteoglycan, and type IV collagen. Plasmin can also indirectly degrade ECM via activation of MMPs. Thus, by impairing the plasminogen activating systems, *PAI-1* can alter organ fibrogenesis^22^,^23^. Recent investigations proposed that *PAI-1* playing a critical role in both acute and chronic hepatic inflammation, and hepatic fibrosis was potently protected by knock-out of *PAI-1*. Further, suggesting that this protective effect could be mediated mostly by ECM resolution by MMPs, which are indirectly inhibited by *PAI-1*^22^. However, our investigation has the limitation of observation of mild therapeutic effects of kupffer cell hyperplasia/proliferation in the α-tocopherol-treated animals and fatty degeneration in the liver of β-carotene-treated animals. This could be due to dosage levels, treatment duration or severity of damage induced by Ang II, and could be resolved by standardising the dosage levels and adjusting the treatment duration.

In addition, expression of CK-19 was clearly suggestive of ductal proliferation. Further, expression of caspase 3 and p53 was also suggestive of apoptosis of hepatocytes in the Ang II-treated experimental *Apoe*^*−/−*^ mice showing evidence of hepatic damage. We also convincingly showed that the expressions of CK-19, caspase 3 and p53 were markedly controlled by β-carotene treatment in the *Apoe*^*−/−*^ mice reflecting the beneficial role of antioxidants^11^,^18^ against hepatic damage.

In summary, our current investigation suggests that subcutaneous Ang II administration induces hepatic damage via the fibrinolysis pathway by the up-regulation of *PAI-1*, activation of hyperplasia of kupffer cells, degeneration of hepatocytes and recruiting inflammatory monocytes/macrophages. In addition, severe sinusoidal dilatations along with haemorrhages were noticed in the liver vascular region indicating the likely induction of portal hypertension by Ang II infusion. Further, α-tocopherol and β-carotene treatment showed a comprehensive effect to repair the hepatic pathology by controlling the recruitment of kupffer cells, inflammatory monocytes/macrophages and controlling the mediator of plasminogen activator system. However, the observation of fatty degeneration in the β-carotene treatment group warrants further extensive investigation to therapeutically utilize these molecules in liver disease^24^.

## Additional Information

**How to cite this article**: Gopal, K. *et al.* Attrition of Hepatic Damage Inflicted by Angiotensin II with α-Tocopherol and β-Carotene in Experimental Apolipoprotein E Knock-out Mice. *Sci. Rep.* **5**, 18300; doi: 10.1038/srep18300 (2015).

## Figures and Tables

**Table 1:** Forward and reverse oligo primer sequence of various candidates genes of mouse used in the present study.

Gene Name	Forward primer	Reverse primer
ICAM1	GACCCAGCTCTCAGCAGTG	CTGGCCTCGGAGACATTAGA
VCAM1	CCACTTGTGCATGGGAGAC	TTTCCCGGTATCTTCAATGG
MCP	CCCACTCACCTGCTGCTACT	TCTGGACCCATTCCTTCTTG
MCSF	CCAAGAACTGCAACAACAGC	GAGAATCATCCCAAGCCAAG
MMP2	GTTGCAACCTCTTTGTGCTG	TGATCTGGTTCTTGTCCCACT
MMP9	GGCCGCTCGGATGGTTAC	TCGCGTCCACTCGGGTAG
MMP12	TGCTCCCATGAATGACAGTGAA	TCCAGTTGCCCAGTTGCTTCTAG
tPA	AGGTCACAGTCCAAGCAATG	GGGTACAGTCTGACGTGAGC
uPA	CCATGCTGACCGTCCTCTCT	CCTGGTCTATAGCATCCTGAG
PAI	GGAGGGCACAACACTTTCAT	GGCCACCATTTGATCTGTCT
uPAR	TGCCGGGGACCAATGAATCAG	CCGTGGCAGCAGGAGACAGAG
PPARα	GCAGTGCTGGCTGGCTACCTTCAAA	TGGGCTACATCCTCGACTCCT
PPAR β	TAGAAGCCATCCAGGACACC	CCGTCTTCTTTAGCCACTGC
PPARy	GGCGAGGGCGATCTTGACAGGAA	CGGATGGCCACCTCTTTGCTCTG
Rn1	TGGGGCGTATTCACAAAGAG	GAAGGTCTGGGGTGGGGTAC

**Table 2:** Liver enzymes of AST and ALT activity levels in peripheral blood of Angiotensin II-infused, and α-tocopherol- and β-carotene-treated *Apoe* ^*−/−*^ mice.

	Experimental Group	Average AST Activity [IUL–1]	P value Vs Control	P value Vs Ang II
AST activity in different treatment groups compared with controls	Control	30.3 ± 6.51		
	Angiotensin II	49.0 ± 11.53	0.01*	No significance
	α-tocopherol	44.7 ± 14.22	0.14	
	β-carotene	34.0 ± 3.61	0.3	
	Treatment control†	66.0 ± 5.29	0.02*	
	**Experimental Animal Group**	**Average ALT Activity [IUL–1]**	**P value Vs Control**	**P value Vs Ang II**
ALT activity in different treatment groups compared with controls	Control	42.33 ± 2.52		
	Angiotensin II	48.00 ± 7.21	0.2	No significance
	α-tocopherol	45.00 ± 7.07	0.43	
	β-carotene	17.50 ± 2.12	0.04*	
	Antioxidants control†	58.67 ± 2.52	0.02*	0.04*

^†^Angiotensin-II treated animals were fed without antioxidants enrichment.

*P values of < 0.05 were considered significant.

**Table 3:** Liver enzymes of AST/ALT ratio in Angiotensin II infused, and α-tocopherol and β-carotene treated*Apoe*^*−/−*^ mice.

Experimental Group	Average AST Activity(IUL-1)	Average ALT Activity(IUL-1)	Ratio of AST/ALT
Control Animal	30.3 ± 6.51	42.33 ± 2.52	0.72
Angiotensin II	49.0 ± 11.53	48.00 ± 7.21	1.02
α-tocopherol	44.0 ± 14.22	45.00 ± 7.07	0.97
β-carotene	34.0 ± 3.61	17.50 ± 2.12	1.94
Antioxidants Control*	66.0 ± 5.29	58.67 ± 2.52	1.12

^*^Angiotensin-II treated animals were fed without antioxidants enrichment.

**Table 4:** Fold changes of candidate genes mRNA level in Ang-II infused, and α-tocopherol and β-carotene treated *Apoe*^*−/−*^ mice.

	Average Fold Expression in Liver		
Reporter Gene	Ang II	α-tocopherol	β-carotene
ICAM-1	1.2*	9.9	6.1
VCAM-1	3.1*	8.1	3.3
M-CSF	56.1*	2*	1.4
MMP-2	14.1*	9.8	12.9
MMP-3	2*	3.1*	6.7
MMP-9	3.8*	19.9	3.1*
MMP12	11.7*	4.2	2.6*
PPAR-α	3.8*	2.1*	2.8*
PPAR-β	4.7*	7.2	2
PPAR-γ	8.5*	23.4*	55.6*
tPA	1.9*	3.6	3.3*
uPA	1*	4.9	1.5
PAI-1	42.8	13.6	19.3

^*^Denotes gene down regulation.

The mRNA expression level is represented as fold.
